# Supplementary material for: Women during Lactation Reduce Their Physical Activity and Sleep Duration Compared to Pregnancy
Source: Int J Environ Res Public Health. 2022 Sep 6;19(18):11199. doi: 10.3390/ijerph191811199 (PMC9517522; doi:10.3390/ijerph191811199)
Supplement: Supplementary file 1 [file ijerph-19-11199-s001.zip › ijerph-1887478-supplementary file S1.pdf]

Facultad de Medicina | UAM Universidad Autónoma de Madrid

Salud materna y su relación con la actividad física, el sueño y el capital psicológico

¡Bienvenida!

A continuación, le planteamos unas preguntas para conocer si su actividad física, su calidad de sueño y sus hábitos de vida se asocian con su salud gestacional o materna. Se estima que se responde en unos 20 minutos.
Sea lo más sincera y realista posible. En las preguntas que lo requieran, tome como referencia el último mes.
La información será tratada de manera confidencial y anónima. Su participación es voluntaria y puede terminarla en cualquier momento. Asimismo, puede plantear todas sus dudas respecto a la investigación a: ***david.ramiro@uam.es*** **y** ***silvia.arribas@uam.es***

Pulsando "Aceptar" acepta participar voluntariamente en este estudio y comprende la información mencionada en los párrafos anteriores.

¡Gracias por participar!

Facultad de Medicina | UAM Universidad Autónoma de Madrid

Salud materna y su relación con la actividad física, el sueño y el capital psicológico

Características generales

* 1. ¿Qué edad tienes?

* 2. ¿Estas embarazada?

- ◯ Sí
- ◯ Estoy en el periodo de lactancia

Facultad de Medicina | UAM Universidad Autónoma de Madrid

Salud materna y su relación con la actividad física, el sueño y el capital psicológico

3. Escribe la semana de gestación (*y si lo supieras, el día*) en la que te encuentras.

Semanas:

Día (escribe "0" en caso de no recordarlo):

4. Marca los apoyos con los que considera que cuenta:

- ☐ Pareja
- ☐ Padres, hermanos u otros familiares
- ☐ Amigos cercanos
- ☐ Otros

Salud materna y su relación con la actividad física, el sueño y el capital psicológico

5. Escribe los días de lactancia que llevas

Días

6. ¿En que semana dio a luz?

Semana:

Dia *(escribe "0" en caso de no recordarlo)*:

7. Marca los apoyos con los que considera que cuenta:

- ☐ Pareja
- ☐ Padres, hermanos u otros familiares
- ☐ Amigos cercanos
- ☐ Otros

8. ¿Es tu primer niño?

◯ Si ◯ No

9. ¿Cuántas **semanas** diste lactancia materna sin ningún otro complemento o formula? *Si no tiene otro niño escriba "0"*

* 10. ¿Cómo de segura te sientes para completar 4 meses de lactancia materna exclusiva?

Muy insegura — Muy segura

* 11. Como de verdad consideras la siguiente afirmación: *¿Me parece bien no saber exactamente la cantidad de leche que recibe mi bebé al ser amamantado?*

Nada verdad — Exactamente opino así

12. ¿Qué tipo de alimentación está dando?

- ◯ Lactancia materna exclusiva
- ◯ Lactancia materna y formula
- ◯ Formula
- ◯ Otras

Salud materna y su relación con la actividad física, el sueño y el capital psicológico

* 13. Su embarazo fue:

- Espontaneo
- Mediante Técnica de Reproducción Asistida

* 14. Nivel de estudios alcanzado

- Sin estudios
- Primarios
- Educación Secundaria, FP de grado medio o similar
- Bachillerato, FP de grado superior o similar
- Licenciatura, Grado o similar
- Estudios de postgrado (master, doctorado o similar)

* 15. ¿Su pais de nacimiento se encuentra?

- Europa - España
- Europa - No España
- África
- Norte-América *(Incluye México y Canadá)*
- Centro-América
- Sur-América
- Asia
- Australia

* 16. Nacionalidad

- Española
- No Española

* 17. Estado civil

- Casada
- Soltera
- En pareja
- Otro

* 18. Situación laboral actual

- Sin trabajo, ama de casa, en paro o pensionista
- Empleada en activo
- De baja
- Estudiante
- Otros

* 19. Nivel económico mensual, *considera los ingresos de su núcleo familiar donde el bebe se va a desarrollar*.

- Sin ingresos
- Menos de 1000€
- Entre 1000 y 2500€
- Entre 2500 y 4000€
- Mas de 4000€

* 20. Consumo de vino/cervezas o similares actualmente

- Nunca
- De forma esporádica *(algún fin de semana)*
- A diario
- Otros

* 21. Consumo de ron/vodka/licores o similares actualmente

- Nunca
- De forma esporádica *(algún fin de semana)*
- A diario
- Otros

* 22. Consumo de tabaco, actual

- No fumo, ni he fumado nunca
- No fumo, pero he fumado hace mas de 2 años
- No fumo, pero he fumado hace menos de 2 años
- Fumo

Salud materna y su relación con la actividad física, el sueño y el capital psicológico

23. Aproximadamente, ¿Cuántos...

cigarrillos/día?

años?

Salud materna y su relación con la actividad física, el sueño y el capital psicológico

Actividad Física

Por favor lea atentamente las preguntas y marque la respuesta que mejor se adapte a lo que haces de forma cotidiana de forma sincera. Recuerda que no hay respuestas ni malas ni buenas.

* 24. Durante este mes, cuando NO está en el trabajo, ¿Cuánto tiempo pasas normalmente...?

	Ninguno	Menos de media hora al día	De media a casi 1 hora al día	De 1 a casi 2 horas al día	De 2 a casi 3 horas al día	3 o más horas al día
Preparando comidas (cocinar, poner la mesa, lavar los platos...)	○	○	○	○	○	○
Vistiendo, bañando, dando de comer a niños **mientras está sentada**	○	○	○	○	○	○
Vistiendo, bañando, dando de comer a niños **mientras está de pie**	○	○	○	○	○	○
Jugando con niños **mientras está sentada o de pie**	○	○	○	○	○	○
Jugando con niños **mientras está andando o corriendo**	○	○	○	○	○	○
Llevando niños en brazos	○	○	○	○	○	○
Cuidando a una persona mayor	○	○	○	○	○	○
Sentada usando el ordenador o escribiendo, cuando no está en el trabajo	○	○	○	○	○	○
Jugando con mascotas	○	○	○	○	○	○
Haciendo las tareas de la casa (hacer la cama, hacer la colada, planchando, ordenando)	○	○	○	○	○	○
Comprando (comida, ropa u otros artículos)	○	○	○	○	○	○
Realizando una limpieza intensa (pasando la aspiradora, fregando, barriendo, limpiando las ventanas)	○	○	○	○	○	○
Sentada mientras come, tanto en casa como en un bar/restaurante	○	○	○	○	○	○
Empujando un carrito de la compra y/o carrito de bebe o silla de ruedas	○	○	○	○	○	○

* 25. Durante este mes, cuando NO está en el trabajo, ¿Cuánto tiempo pasas normalmente...?

	Ninguno	Menos de media hora al día	De media a casi 2 hora al día	De 2 a casi 4 horas al día	De 4 a casi 6 horas al día	6 o más horas al día
Viendo la televisión o vídeos	○	○	○	○	○	○
Sentada leyendo, hablando o con el teléfono, **cuando no está en el trabajo**	○	○	○	○	○	○

* 26. Yendo a lugares, durante este mes, ¿Cuánto tiempo pasas normalmente...?

	Ninguno	Menos de media hora al día	De media a casi 1 hora al día	De 1 a casi 2 horas al día	De 2 a casi 3 horas al día	3 o más horas al día
Caminando **lentamente** para ir a sitios (como a la parada de autobús, al trabajo, de visita). *No por placer ni para hacer ejercicio físico*	○	○	○	○	○	○
Caminando **deprisa** para ir a sitios (como a la parada de autobús, al trabajo, de visita). *No por placer ni para hacer ejercicio físico*	○	○	○	○	○	○
Conduciendo o montando en un coche o autobús	○	○	○	○	○	○

* 27. Por placer o para hacer ejercicio físico, durante este mes, ¿Cuánto tiempo pasas normalmente...?

	Ninguno	Menos de media hora al día	De media a casi 1 hora al día	De 1 a casi 2 horas al día	De 2 a casi 3 horas al día	3 o más horas al día
Caminando **lentamente** por placer o para hacer ejercicio físico	○	○	○	○	○	○
Caminando **deprisa** por placer o para hacer ejercicio físico	○	○	○	○	○	○
Caminando deprisa cuesta arriba por placer o para hacer ejercicio físico	○	○	○	○	○	○

* 28. Durante este mes, ¿Cuánto tiempo pasas normalmente...?

	Ninguno	Menos de media hora al día	De media a casi 1 hora al día	De 1 a casi 2 horas al día	De 2 a casi 3 horas al día	3 o más horas al día
Caminando con cierta velocidad	○	○	○	○	○	○
En clase de ejercicio prenatal	○	○	○	○	○	○
Nadando	○	○	○	○	○	○
Bailando	○	○	○	○	○	○
Otras actividades que requieran ejercicio físico *(Yoga, Pilates, etc.)*	○	○	○	○	○	○

* 29. En el trabajo, durante este mes, ¿Cuánto tiempo pasas normalmente...?

	Ninguno *(o soy ama de casa, desempleada o no puedo trabajar)*	Menos de media hora al día	De media a casi 2 hora al día	De 2 a casi 4 horas al día	De 4 a casi 6 horas al día	6 o más horas al día
Sentada en el trabajo o en clase	○	○	○	○	○	○
De pie o caminando lentamente mientras carga con cosas (*que pesen más que dos botellas de agua de 1.5L*)	○	○	○	○	○	○
De pie o caminando lentamente en el trabajo sin cargar nada	○	○	○	○	○	○
Caminado deprisa mientras carga con pesos (*que pesen más que dos botellas de agua de 1.5L*)	○	○	○	○	○	○
Caminando deprisa en el trabajo sin cargar nada	○	○	○	○	○	○

Salud materna y su relación con la actividad física, el sueño y el capital psicológico

Calidad del Sueño

Las siguientes preguntas tratan de tus hábitos de sueño, solo durante el pasado mes. Tus respuestas deben indicar los mas sincera y adecuadamente posible qué paso el pasado mes. Recuerda que no hay respuestas buenas ni malas.

* 30. Durante el pasado mes, ¿A que hora te has ido a la cama? *(recuerda que de 00:00 a 12:00 sería a.m. y de 12:01 a 23:59 p.m.)*

Hora

Hora a. m./p. m.

hh mm -

* 31. Durante el pasado mes, ¿Cuánto tiempo **en minutos** tardaste en quedarte dormida?

* 32. Durante el pasado mes, ¿A que hora te has despertado? *(recuerda que de 00:00 a 12:00 sería a.m. y de 12:01 a 23:59 p.m.)*

Hora

Hora a. m./p. m.

hh mm -

* 33. Durante el pasado mes, ¿Cuántas **horas al día** has dormido realmente? *(esto puede ser distinto al numero de horas que has permanecido en la cama)*

* 34. Durante el pasado mes, ¿Cuántas veces has tenido problemas para quedarte dormida debido a...?

	No durante el pasado mes	Menos de 1 vez a la semana	1 o 2 veces a la semana	3 o mas veces a la semana
No quedarme dormida en los primeros 30 minutos	◯	◯	◯	◯
Despertarme en mitad de la noche o muy temprano en la mañana	◯	◯	◯	◯
Tener que usar el baño con frecuencia	◯	◯	◯	◯
Respirar con dificultad	◯	◯	◯	◯
Toser o roncar fuertemente	◯	◯	◯	◯
Sentir demasiado frío	◯	◯	◯	◯
Sentir demasiado calor	◯	◯	◯	◯
Tener malos sueños o pesadillas	◯	◯	◯	◯
Dolor	◯	◯	◯	◯
Otras razones	◯	◯	◯	◯

* 35. Durante el pasado mes, **en general**, ¿Cómo calificaría la calidad de su sueño?

◯ Muy buena ◯ Bastante buena ◯ Bastante mala ◯ Muy mala

* 36. Durante el pasado mes, ¿Cuántas veces has...

	No durante el pasado mes	Menos de 1 vez a la semana	1 o 2 veces a la semana	3 o mas veces a la semana
Tomado medicación para ayudarte a dormir *(prescrita o por tu cuenta)?*	◯	◯	◯	◯
Tenido problemas para estar despierta mientras conducías, comías, o participando en actividades sociales?	◯	◯	◯	◯

* 37. Durante el pasado mes, ¿Te ha supuesto un problema mantener el entusiasmo en hacer cosas?

◯ Nada en absoluto ◯ Sólo un pequeño problema ◯ Un poco ◯ Un gran problema

Salud materna y su relación con la actividad física, el sueño y el capital psicológico

Capital Psicológico

Por favor, responde como de cierto o incierto son las siguientes afirmaciones, según tu punto de vista.

* 38. Marca la respuesta que mejor represente su opinión acerca de las siguientes afirmaciones

	Totalmente incierto	Apenas incierto	Más bien cierto	Totalmente cierto
En tiempos difíciles suelo esperar lo mejor	○	○	○	○
Consigo alcanzar mis metas aunque haya obstáculos	○	○	○	○
Pienso que mi vida tiene sentido	○	○	○	○
Tengo confianza en que podría manejar eficazmente acontecimientos inesperados	○	○	○	○
Cuando pienso en mi futuro siempre soy optimista	○	○	○	○
Aunque las cosas vayan mal, no me rindo	○	○	○	○
Creo que cada día es valioso	○	○	○	○
Venga lo que venga, por lo general, soy capaz de manejarlo	○	○	○	○
En general, espero que me ocurran más cosas buenas que malas	○	○	○	○
Soy capaz de tomar decisiones difíciles	○	○	○	○
Siento que mi vida tiene valor y merece la pena	○	○	○	○
Puedo resolver la mayoría de los problemas si me esfuerzo lo necesario	○	○	○	○

Salud materna y su relación con la actividad física, el sueño y el capital psicológico

Salud General (SF-36)

Las siguientes preguntas valoran los estados tanto positivos como negativos de tu salud. Responde lo mas honestamente posible.

* 39. En general, diría que tu salud es:

- ◯ Excelente
- ◯ Muy buena
- ◯ Buena
- ◯ Regular
- ◯ Mala

* 40. ¿Cómo dirías que es tu salud actual, comparada con la de hace un año?

- ◯ Mucho mejor ahora que hace un año
- ◯ Algo mejor ahora que hace un año
- ◯ Más o menos igual que hace un año
- ◯ Algo peor ahora que hace un año
- ◯ Mucho peor ahora que hace un año

* 41. Las siguientes preguntas se refieren a actividades o cosas que podrías hacer en un día normal. Tu salud:

	Sí, me limita mucho	Sí, me limita un poco	No, no me limita nada
¿Te limita para hacer esfuerzos intensos *(correr, levantar objetos pesados, o participar en deportes)*?	◯	◯	◯
¿Te limita para hacer esfuerzos moderados *(mover una mesa, pasar la aspiradora, jugar a los bolos o caminar +1h)*?	◯	◯	◯
¿Te limita para coger o llevar la bolsa de la compra?	◯	◯	◯
¿Te limita para subir **varios pisos** por la escalera?	◯	◯	◯
¿Te limita para subir **un solo piso** por la escalera?	◯	◯	◯
¿Te limita para agacharte o arrodillarte?	◯	◯	◯
¿Te limita para caminar un kilómetro o más?	◯	◯	◯
¿Te limita para caminar varias calles?	◯	◯	◯
¿Te limita para caminar unos 100 metros?	◯	◯	◯
¿Te limita para bañarte o vestirte por ti misma?	◯	◯	◯

* 42. Las siguientes preguntas se refieren a problemas en su trabajo o en sus actividades diarias, durante las ultimas 4 semanas:

	Sí	No
¿Tuviste que reducir el tiempo dedicado al trabajo o a sus actividades cotidianas a causa de su salud física?	○	○
Hiciste menos de lo que hubieras querido hacer, a causa de su salud física?	○	○
¿Tuviste que dejar de hacer algunas tareas en su trabajo o en sus actividades cotidianas, a causa de su salud física?	○	○
¿Tuviste dificultad para hacer su trabajo o sus actividades cotidianas *(te costó más de lo normal)*, a causa de su salud física?	○	○
¿Tuviste que reducir el tiempo dedicado al trabajo o a sus actividades cotidianas a causa de algún problema emocional *(como estar triste, deprimida, o nerviosa)*?	○	○
¿Hiciste menos de lo que hubieras querido hacer a causa de algún problema emocional *(como estar triste, deprimida, o nerviosa)*?	○	○
¿No hiciste tu trabajo o sus actividades cotidianas tan cuidadosamente como de costumbre, a causa de algún problema emocional *(como estar triste, deprimida, o nerviosa)*?	○	○

* 43. Durante las últimas 4 semanas, ¿hasta qué punto tu salud física o los problemas emocionales han dificultado tus actividades sociales habituales con la familia, los amigos, los vecinos u otras personas?

- ◯ Nada ◯ Un poco ◯ Regular ◯ Bastante ◯ Mucho

* 44. ¿Tuviste dolor en alguna parte del cuerpo durante las 4 últimas semanas?

- ◯ No, ninguno ◯ Sí, muy poco ◯ Sí, un poco ◯ Sí, moderado ◯ Si, mucho ◯ Sí, muchísimo

* 45. Durante las últimas 4 semanas, ¿hasta qué punto el dolor le ha dificultado su trabajo habitual *(incluido el trabajo fuera de casa y las tareas domésticas)*?

- ◯ Nada ◯ Un poco ◯ Regular ◯ Bastante ◯ Mucho

* 46. Las siguientes preguntas se refieren a cómo te has sentido y como te han ido las cosas **durante las 4 últimas semanas**. Responde, ¿Cuánto tiempo...

	Siempre	Casi siempre	Muchas veces	Algunas veces	Sólo alguna vez
Te sentiste llena de vitalidad?	◯	◯	◯	◯	◯
Estuviste muy nerviosa?	◯	◯	◯	◯	◯
Te sentiste tan baja de moral que nada podía animarte?	◯	◯	◯	◯	◯
Te sentiste calmada y tranquila?	◯	◯	◯	◯	◯
Tuviste mucha energía?	◯	◯	◯	◯	◯
Te sentiste desanimada y triste?	◯	◯	◯	◯	◯
Te sentiste agotada?	◯	◯	◯	◯	◯
Te sentiste feliz?	◯	◯	◯	◯	◯
Te sentiste cansada?	◯	◯	◯	◯	◯
¿Con qué recurrencia la salud física o los problemas emocionales te han dificultado tus actividades sociales *(como visitar a amigos o familiares)*?	◯	◯	◯	◯	◯

* 47. Por favor, di si te parece cierta o falsa cada una de las siguientes frases:

	Totalmente cierta	Bastante cierta	No lo sé	Bastante falsa	Totalmente falsa
Creo que me pongo enferma más fácilmente que otras personas					
Estoy tan sana como cualquiera					
Creo que mi salud va a empeorar					
Mi salud es excelente					

Salud materna y su relación con la actividad física, el sueño y el capital psicológico

Gestación y Maternidad

* 48. Tu gestación o lactancia:

- ◯ Soy madre en periodo de lactancia de un bebe
- ◯ Soy madre en periodo de lactancia de mas de 1 bebe
- ◯ Soy gestante de un embrión o feto
- ◯ Soy gestante de más de un embrión o feto

Salud materna y su relación con la actividad física, el sueño y el capital psicológico

49. El sexo fetal es:

- ◯ Varon
- ◯ Hembra
- ◯ Aún no lo se

Salud materna y su relación con la actividad física, el sueño y el capital psicológico

50. Los sexos fetales son:

- ◯ Varones
- ◯ Hembras
- ◯ Varones y Hembras
- ◯ Aún no lo se

51. El sexo del bebé es:

- Varón
- Hembra
- Otros

Salud materna y su relación con la actividad física, el sueño y el capital psicológico

52. Los sexos de los bebes son:

- ◯ Varón
- ◯ Hembra
- ◯ Varones y Hembras
- ◯ Otros

Salud materna y su relación con la actividad física, el sueño y el capital psicológico

* 53. Marca si ha padecido o padece alguna de las siguientes complicaciones del embarazo o la lactancia *(siempre y cuando lo recuerdes, de lo contrario, indique" no")*

	Sí	No
Amenaza de parto prematuro	○	○
Parto prematuro	○	○
Rotura prematura de membranas	○	○
Diabetes Gestacional	○	○
Hipertensión inducida por el embarazo	○	○
Preeclampsia	○	○
Corioamnionitis	○	○
Colectasis intrahepatica	○	○
Crecimiento intrauterino retardado	○	○
Pequeño para la edad gestacional	○	○
Mastitis	○	○
Dolor en la mama o pezón	○	○
Grietas	○	○

* 54. Marca si padece alguna de las siguientes dolencias *(de lo contrario, indique" no")*

	Sí	No
Hipertensión	◯	◯
Colesterol elevado	◯	◯
Obesidad	◯	◯
Triglicéridos elevados	◯	◯
Diabetes Mellitus	◯	◯
Asma	◯	◯
Cáncer	◯	◯
Enfermedades autoinmunes *(como VIH, lupus, etc)*	◯	◯
Depresión u otros trastorno emocional	◯	◯

Salud materna y su relación con la actividad física, el sueño y el capital psicológico

Conoce al grupo de Investigación

¡Gracias por llegar hasta aquí!

El grupo de investigación FOSCH (*Food Oxidative Stress and Cardiovascular Health*) tiene como uno de sus objetivos la prevención de las enfermedades cardio-metabólicas a través de la alimentación, especialmente en etapas tempranas de la vida.

Los resultados del estudio se publicarán en revistas científicas y en nuestras redes sociales (*@FoschGroup*).

Si quiere seguir colaborando con nosotros en futuros estudios, rellene el siguiente campo para que nos pongamos en contacto contigo.

¡Muchas gracias de nuevo!
Grupo de Investigación FOSCH

55. Correo electrónico
